# dSir2 deficiency in the fatbody, but not muscles, affects systemic insulin signaling, fat mobilization and starvation survival in flies

**DOI:** 10.18632/aging.100435

**Published:** 2012-03-10

**Authors:** Kushal Kr. Banerjee, Champakali Ayyub, Samudra Sengupta, Ullas Kolthur-Seetharam

**Affiliations:** Tata Institute of Fundamental Research Department of Biological Sciences, Tata Institute of Fundamental Research, Colaba, Mumbai 400 005, India

**Keywords:** Sir2, fat metabolism, starvation, survival, fatbody, muscles, insulin signaling

## Abstract

Sir2 is an evolutionarily conserved NAD^+^ dependent protein. Although, SIRT1 has been implicated to be a key regulator of fat and glucose metabolism in mammals, the role of Sir2 in regulating organismal physiology, in invertebrates, is unclear. Drosophila has been used to study evolutionarily conserved nutrient sensing mechanisms, however, the molecular and metabolic pathways downstream to Sir2 (dSir2) are poorly understood. Here, we have knocked down endogenous dSir2 in a tissue specific manner using gene-switch gal4 drivers. Knockdown of dSir2 in the adult fatbody leads to deregulated fat metabolism involving altered expression of key metabolic genes. Our results highlight the role of dSir2 in mobilizing fat reserves and demonstrate that its functions in the adult fatbody are crucial for starvation survival. Further, dSir2 knockdown in the fatbody affects dilp5 (insulin-like-peptide) expression, and mediates systemic effects of insulin signaling. This report delineates the functions of dSir2 in the fatbody and muscles with systemic consequences on fat metabolism and insulin signaling. In conclusion, these findings highlight the central role that fatbody dSir2 plays in linking metabolism to organismal physiology and its importance for survival.

## INTRODUCTION

Studies from different model organisms have shown that nutrient sensing factors or pathways [1, 2] such as AMP-activated kinase (AMPK) [3], insulin-IGF signaling (IIS) [4, 5], target of rapamycin (TOR) [6] and Sirtuins (Sir2-like) [7, 8] are key players in maintaining metabolic homeostasis. Further, the response of an organism to a metabolic stress, such as starvation, is largely dependent on its capacity to derive energy from stored reserves like triglyceride (lipid) [9, 10] and glycogen (glucose) [11, 12]. Genetic perturbations that impinge on lipid metabolism have been implicated as one of the causes of metabolic deregulation, and often associated with obesity and type II diabetes [13-16].

Sirtuins or Sir2-like proteins are NAD+-dependent enzymes that link cellular physiology with metabolic changes [7, 17]. Sir2 was identified as a key determinant of longevity in *Saccharomyces cerevisiae* and its dependence on NAD+ for its activity is crucial for its ability to link calorie/dietary restriction (CR/DR) with lifespan extension [18, 19]. Although, initial observations in *Caenorhabditis elegans* [20] and *Drosophila melanogaster* [21] indicated the evolutionary significance of Sir2 in extending lifespans, recent reports have questioned these findings in metazoans [22, 23, 24]. More importantly, in the absence of a clear understanding of the evolutionary conservation of molecular functions of Sir2 in metazoans, its roles in regulating organismal physiology and survival are still not well appreciated. In this regard, studies that dissect out molecular functions of Sir2 will provide insights into its role in response to calorie restriction and aging.

Although, it is intuitive to expect Sir2 to maintain metabolic homeostasis, across species, only mammalian SIRT1 has been clearly shown to do so [17, 25, 26]. Cell culture studies clearly indicated the role of SIRT1 in glucose and fat metabolism and recent papers have highlighted its importance in metabolically relevant tissues such as the liver and muscles [26-36]. Further, the contributions of SIRT1 functions in different tissues in maintaining organismal physiology are being appreciated only recently [36, 37]. However, it is still unclear if these functions of SIRT1 are evolutionarily conserved in lower organisms. Therefore, there is a need to study the roles of Sir2 orthologs in mediating systemic changes, and those emanating from metabolic tissues, to provide a comprehensive understanding of Sir2 biology. Although, studies in mammals provide clear insights into the ability of SIRT1 in metabolically relevant tissues to affect organismal physiology [36, 37], the relevance under altered nutrient conditions is not known. Importantly, it is unclear whether the ability of Sir2 in different tissues to maintain metabolic homeostasis has any bearing on organismal survival.

The fruit fly *Drosophila melanogaster* has been successfully used for studying many molecular factors that link dietary or metabolic changes to organismal physiology [38]. The molecular mechanisms downstream to *dSir2* and its ability to regulate metabolism are poorly appreciated in flies. *dSir2* was previously shown to genetically interact with *rpd3* (histone deacetylase) and mediate lifespan extension [21]. However, this does not provide a functional link with the metabolic functions (if any) of *dSir2* [39]. *dSir2* has also been shown to interact with Dmp53 and is implicated in p53-dependent functions [40, 41]. Except for a very recent study in larvae, its role in regulating metabolism in adult flies is still unclear [42]. Importantly, the tissue specific role of dSir2 in modulating metabolism and therefore, organismal physiology has not been addressed in adult flies. Given the tissue complexity and conservation of metabolic pathways, Drosophila is a useful system to investigate dSir2-dependent alterations in molecular mechanisms, which might have a bearing on organismal survival.

Here, we report the central role of endogenous dSir2 in maintaining metabolic and energy homeostasis. In addition to using backcrossed *dSir2* mutant flies, we have knocked down *dSir2* using gene-switch lines that negate background genetic differences (between control and dSir2 knockdown flies). We show that dSir2 plays a crucial role in fat metabolism and systemic insulin signaling. By knocking down dSir2 in the muscles and fatbody, we have compared its functions in these tissues to maintain metabolic homeostasis and mediate organismal survival in response to starvation. These findings not only establish *Drosophila* as a useful system to investigate Sir2 functions, but also show that dSir2 is a critical factor in fat mobilization from the fatbody during starvation. Our results highlight the key role that dSir2 plays in the adult fatbody in bringing about systemic changes in physiology that determine the ability of an organism to cope with metabolic stress. Importantly, contrasting effects of *dSir2* deficiency in the fatbody and muscles signify the tissue dependent roles of dSir2 in organismal survival.

## RESULTS

### dSir2 affects starvation survival in flies

**Figure 1:**
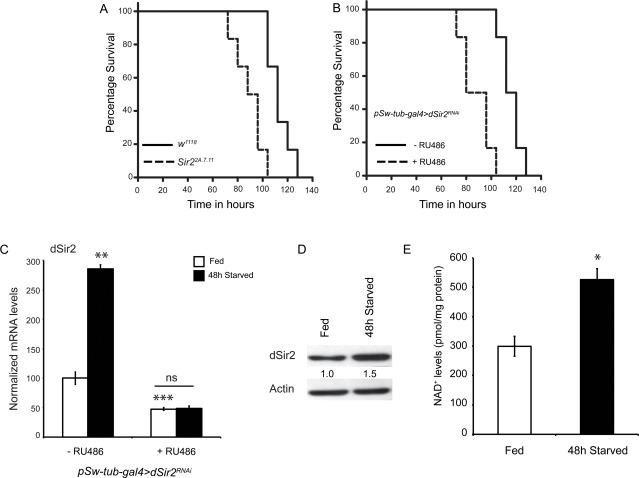
dSir2 activity increases in response to starvation and its absence decreases starvation resistance (**A**) Starvation survival of *dSir2* mutants (*Sir2^2A.7.11^*) (p < 0.001) and (**B**) whole body *dSir2^RNAi^* (+ RU486) (p < 0.001), with respective controls (n = 60). (**C)** *dSir2* transcript levels increase in response to 48-hours starvation in control (- RU486) but not in whole body *dSir2^RNAi^*(+ RU486) flies (n = 8/24). (**D**) Increase in *dSir2* protein levels in control flies in response to 48-hours starvation. (**E**) Increase in NAD^+^ levels in response to 48-hours starvation in control flies (n = 36). 200 μM RU486 was used to knockdown *dSir2* expression in*pSw-tub-gal4>dSir2^RNAi^* flies. Log Rank was used to plot survival curves and Mantel-Cox test was used for statistical analysis. Student's t-test and ANOVA were used to analyze statistical significance of the data (*, p < 0.05; **, p < 0.01; ***, p < 0.001 or mentioned otherwise).

Starvation, unlike other dietary regimes, is an acute manipulation, which induces nutrient sensing pathways [46]. In addition, it reflects the ability of an organism to maintain metabolic and energy homeostasis [46]. Although, SIRT1 in mammals has been shown to respond to starvation and mediate transcription of genes involved in metabolic homeostasis [27, 34, 47], such a role for Sir2 in invertebrates is not known. Moreover, it is still unclear if absence of Sir2/SIRT1 affects starvation resistance. In order to study the importance of *Drosophila* Sir2 (dSir2) in maintaining metabolic homeostasis, we subjected the flies to nutrient deprivation (starvation). We found that backcrossed *dSir2* mutant (Sir^22A.7.11^) flies (Supplementary Figure 1) were more sensitive to starvation when compared to the controls (Figure 1A and Supplementary Figure 2). In order to confirm this and also negate any potential background effects, we knocked down *dSir2* ubiquitously using the inducible *pSwitch-tub-gal4* driver (Supplementary Figure 1). Knocking down *dSir2* (+ RU486) also led to decreased starvation survival similar to the mutant flies (Figure 1B and Supplementary Figure 2).

SIRT1 activity and expression have been shown to increase during starvation in mammals [48, 49]. To check if starvation resistance in control flies involved an upregulation of *dSir2* expression, we assayed for its transcript and protein levels. *dSir2* expression, both mRNA and protein, was induced in response to starvation in control flies (Figures 1C and 1D). It is important to note that *dSir2^RNAi^* (+ RU486) flies did not show any increase in *dSir2* transcript levels (Figure 1C) upon starvation.

The activity of Sir2-like proteins has been demonstrated to be dependent upon intracellular NAD^+^ levels. Across organisms, increased NAD^+^ levels have been associated with increased activity and functions of *Sir2* proteins [50-54]. To check if in addition to induction of *dSir2* expression, there was an increase in NAD^+^ levels during starvation, we measured NAD^+^ in fed and starved, control and *dSir2^RNAi^* (+ RU486) flies (Figure 1E and Supplementary Figure 2). We found that NAD*^+^* levels increased by 1.8 folds in response to starvation, similarly, in control and *dSir2^RNAi^*(+ RU486) flies (Figure 1E and Supplementary Figure 2). These results indicate that dSir2 expression and activity, *in vivo*, increases during starvation in flies.

### *dSir2* in the fatbody regulates metabolic homeostasis in flies

**Figure 2:**
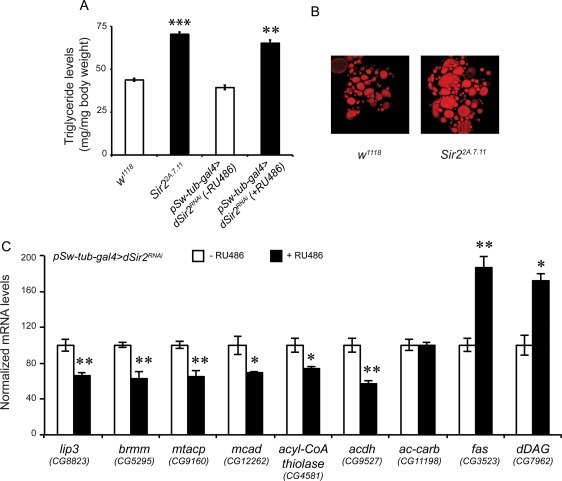
dSir2 regulates fat metabolism (**A**) Total body triglyceride (TAG) in *dSir2* mutants (*Sir2^2A.7.11^*) and whole body *dSir2^RNAi^* (+ RU486) flies, with respective controls (n = 36). (**B**) Oil-Red O staining of fatbodies from control and *Sir2^2A.7.11^*. (**C**) Relative expression of fat metabolism genes *lipase-3 (lip3)*, *brummer (brmm), mitochondrial acyl carrier protein (mtACP), medium chain acyl CoA dehydrogenase (mcad), aceto acetyl CoA thiolase (ACoT), long chain acyl CoA dehydrogenase (lcad), acetyl CoA carboxylase (ACC), fatty acid synthase (fas)* and *diacyl glycerol synthase (dDAG)* (n = 24). 200 μM RU486 was used to knockdown dSir2 expression in *pSw-tub-gal4>dSir2^RNAi^* flies. Student's t-test was used to analyze statistical significance of the data (*, p < 0.05; **, p < 0.01; ***, p < 0.001 or mentioned otherwise).

Abrogated starvation resistance indicated metabolic defects in flies that lacked *dSir2*. To appreciate the importance of dSir2 in maintaining metabolic homeostasis, total glucose and total triglyceride levels were measured in control and backcrossed *dSir2* mutant flies (*Sir2^2A.7.11^*). Interestingly, we found that both were significantly higher in *dSir2* mutants (*Sir2^2A.7.11^*) (Figure 2A and Supplementary Figure 3A). The deregulated fat metabolism and increased triacylglycerol (TAG) levels were corroborated by oil red staining of fatbodies (Figure 2B) in these flies. In order to check if dSir2 was also responsible for regulating metabolism under altered dietary conditions, we fed the control and mutant flies with diets containing variable yeast contents (0.25%, 2.5% and 5.0% with 5.0% sucrose and 8.6% cornmeal). It is important to note that irrespective of the dietary regime, flies that lacked*dSir2* exhibited increased glucose and triglyceride levels (Supplementary Figures 3B and 3C).

When glucose levels were assayed in whole body *dSir2* knockdown flies, we observed decreased levels of glucose, contrary to what was found in the *dSir2* mutant flies (Supplementary Figure 3A). This difference could be due to the complete absence of *dSir2* expression in the mutants, as against a reduced expression in the RNAi flies (Supplementary Figure 1). Importantly, we saw an increase in the TAG levels when *dSir2* was knocked down in the whole body and this effect was consistent with the mutants (Figures 2A and 2B). We did not observe a significant difference in the weights of these flies (Supplementary Figure 3D). To confirm the role of dSIr2 in regulating metabolic output in a bidirectional manner, we overexpressed dSir2 using *pSw-tub-gal4>Sir2^EP2300^*. The flies overexpressing dSir2 exhibited decreased TAG levels (Supplementary Figure 4A). These results clearly showed that an absence of dSir2 leads to metabolic defects, specifically of fat metabolism, under basal conditions and high nutrient conditions.

To investigate the underlying molecular mechanism that renders this phenotype, we quantified the expression of genes involved in fat metabolism by RT-qPCR analyses. From Figure 2C it is evident that in *dSir^2RNAi^*flies (+ RU486), the transcript levels of *brummer* (lipase ATGL), *lipase-3, medium chain acyl-CoA dehydrogenase (MCAD)*, *mitochondrial acyl carrier protein (mtACP), aceto-acetyl-CoA-thiolase (ACoT)* and *long chain acyl-CoA-dehydrogenase (LCAD)* are decreased. We also observed that expression of *fatty acid synthase (fas)* and *diacyl glycerol synthetase (dDAG)* were upregulated in *dSir^2RNAi^* flies (+ RU486) (Figure 2D). Corroborating the importance of dSir2 in the expression of these genes, we observed the opposite effects in the dSir2 overexpression flies (Supplementary Figure 4).

**Figure 3:**
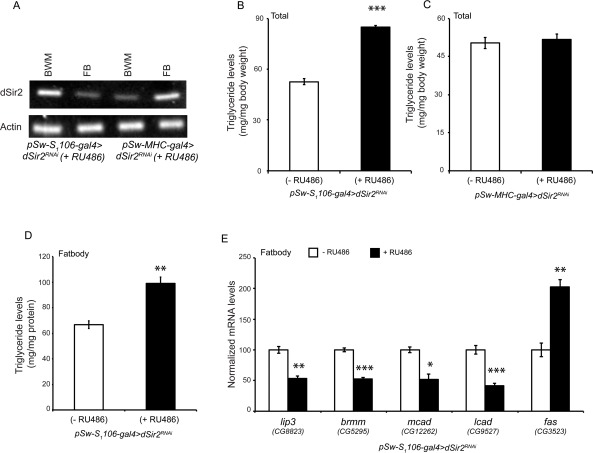
dSir2 in the fatbody, but not in the muscles regulates fat metabolism (**A**) RT-PCR to show knockdown of *dSir2* in the fatbody (FB) of fatbody *dSir2^RNAi^*and body wall with muscles (BWM) of muscle *dSir2^RNAi^,* with respective controls (n = 24). (**B**-**C**) Total body triglyceride levels in (**B**) fatbody *dSir2^RNAi^* flies and (**C**) muscles *dSir2^RNAi^* flies (n = 36). (**D**) Triglyceride levels in the isolated fatbody of fatbody *dSir2^RNAi^* flies (n = 60). (**E**) Relative expression of fat metabolism genes *lipase-3 (lip3)*, *brummer (brmm), medium chain acyl CoA dehydrogenase (mcad), long chain acyl CoA dehydrogenase (lcad)* and *fatty acid synthase (fas)* in fatbody isolated from fatbody *dSir2^RNAi^* flies (n = 24). 200 μM RU486 was used to knockdown *dSir2* expression *pSw-S_1_106-gal4>dSir2^RNAi^* and *pSw-MHC-gal4> dSir2^RNAi^* flies. Student's t-test was used to analyze statistical significance of the data (*, p < 0.05; **, p < 0.01; ***, p < 0.001 or mentioned otherwise).

In order to check if the accumulation of TAGs was due to a failure in mobilization or utilization, we knocked down *dSir2* in a tissue specific manner. While muscles utilize fat, fatbodies are considered equivalent to mammalian liver and adipose tissue. We knocked down *dSir2* in the fatbody using the specific *pSw-S_1_106-gal4* driver, which has previously been reported to be expressed in abdominal and head fatbodies [55, 56]. To knockdown *dSir2* in the muscles, we employed the muscles specific *pSwitch-MHC-gal4* driver [57]. Figure 3A (and Supplementary Figure 5) clearly show tissue specific knockdown of *dSir2* in these flies. It is very interesting to note that fatbody knockdown of *dSir2* led to an increase in TAG levels and mimicked the whole body *dSir^2RNAi^* (+ RU486) flies (Figure 2A). Surprisingly, knocking down *dSir2* in the muscles did not affect TAG levels (Figure 3C), suggesting the importance of dSir2 in fat mobilization.

These results indicated that in the absence of *dSir2* the defects in fat metabolism originate in the fatbodies. The importance of *dSir2* in this tissue was confirmed when we observed increased TAG levels in fatbodies isolated from *fatbody dSir^2RNAi^* (+ RU486) flies (Figure 3B). This increase was also corroborated by reduced expression of fat breakdown genes and an increase in fatty acid synthase (Figure 3E) in the fatbody. These findings show that *dSir2* is important for metabolic homeostasis, and highlight its role in the adult fatbody in regulating fat metabolism. To our knowledge, this is the first study linking *dSir2* to metabolic homeostasis in adult flies, and with the recent findings in larvae [42], highlights its role in regulating fat metabolism in invertebrates. Importantly, from our results, and studies on mammalian SIRT1 [29, 33, 58] an evolutionarily conserved role for *Sir2* (dSir2/SIRT1) in regulating fat metabolism becomes obvious.

### Knockdown of *dSir2* in the fatbody, but not in muscles, affects starvation survival

**Figure 4:**
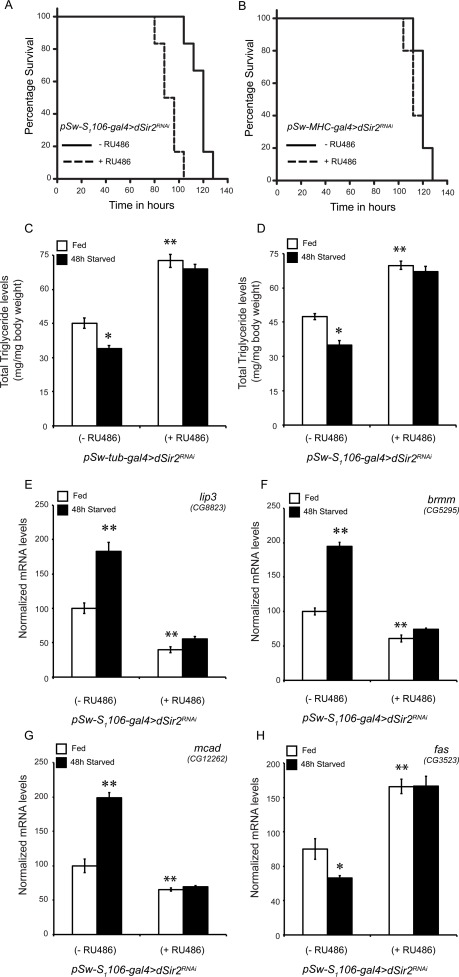
Fatbody dSir2, but not muscle specific dSir2 regulates starvation survival and regulates fat mobilization (**A** and **B**) Starvation survival in (**A**) fatbody *dSir2* knockdown flies (p < 0.001) and (**B**) muscle *dSir2* knockdown flies (non-significant, ns)(n = 60). 200 μM RU486 was used to knockdown *dSir2* expression *pSw-S_1_106-gal4>dSir2^RNAi^* and *pSw-MHC-gal4> dSir2^RNAi^* flies. Log Rank was used to plot survival curves and Mantel-Cox test was used for statistical analysis. (**C**-**D**) Total body triglyceride levels in fed and starved conditions in (**C**) whole body*dSir2^RNAi^*flies, with respective controls (n = 36) and (D) fatbody *dSir2^RNAi^* (n = 36). Relative expression of fat metabolism genes (**E**) *lipase-3* (lip3), (F) *brummer (brmm),* (**G**) *medium chain acyl CoA dehydrogenase (mcad)*, (**H**) *fatty acid synthase (fas)* in fatbody *dSir2* knockdown flies under fed and starved conditions (n = 24). 200 μM RU486 was used to knockdown *dSir2* expression *pSw-S_1_106-gal4>dSir2^RNAi^* and *pSw-MHC-gal4> dSir2^RNAi^* flies. ANOVA was used to analyze statistical significance of the data (*, p < 0.05; **, p < 0.01; ***, p < 0.001 or mentioned otherwise).

Based on the molecular results, we wanted to check if knocking down *dSir2* in specific tissues, such as fatbody and muscles, affected starvation survival. Control and *dSir^2RNAi^* (+ RU486) flies, in both muscles and fatbodies, were assayed for starvation survival. Our results clearly demonstrate that knocking down *dSir2* in the fatbody phenocopied (Figure 4A and Supplementary Figure 6) the ubiquitous *dSir2* knockdown (Figure 1B) and these flies succumbed to starvation earlier than the controls. Interestingly, subjecting *pSwitch-MHC-gal4* (+ RU486) flies to starvation phenocopied control flies (Figure 4B). These results clearly delineate the functions of dSir2 in the two different metabolic tissues in affecting starvation survival.

### Failure to mobilize energy reserves affects starvation survival in fatbody dSir2 knockdown flies

We were intrigued to find that, although, *dSir2*-RNAi and -mutant flies had increased fat storage (Figures 2A and 2B), these flies survived less upon starvation. Tissue specific knockdown of dSir2 in the fatbody and the muscles indicated that these flies had defective fat mobilization and not utilization (Figures 3B to 3D). To check if this was true, we analyzed TAG levels and genes involved in fat metabolism in response to starvation. While control flies showed a significant decrease in TAG levels after 48-hour starvation, whole body *dSir^2RNAi^* (+ RU486) flies had elevated TAG levels and were comparable to their fed condition (Figure 4C). Importantly, the same effect was observed when *dSir2* was knocked down specifically in the fatbody (Figure 4D). Corroborating these results, we found that the expression of genes involved in fat breakdown increased in control flies during starvation, but not in fatbody *dSir^2RNAi^* (+ RU486) flies (Figures 4E to 4G). Surprisingly, we also observed an increase in the expression of fatty acid synthase in fatbody *dSir2* knockdown flies under fed and starved conditions (Figure 4H).

### Fatbody specific knockdown of *dSir2* affects insulin signaling

**Figure 5:**
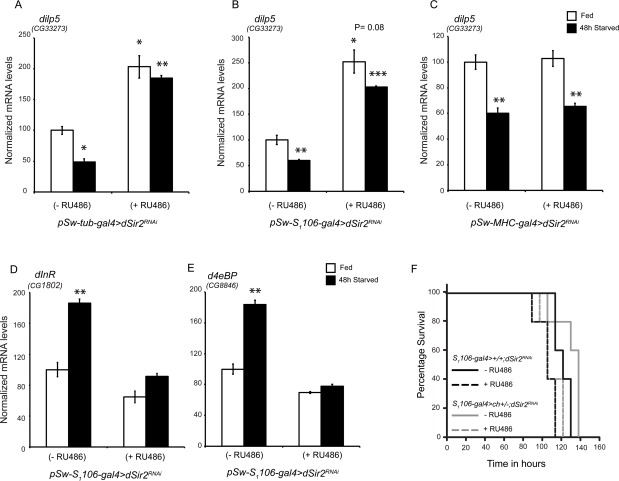
Fatbody dSir2 regulates dilp5 meditated Insulin Signaling (**A**-**C)** Relative *dilp5* levels in the heads of (**A**) whole body *dSir2^RNAi^* (**B**) fatbody *dSir2^RNAi^* and (**C**) muscle *dSir2^RNAi^* flies, with respective controls, under fed and starved conditions (n = 24). Relative expression of (**D**) *dInR* and (E) *d4eBP* in fatbody *dSir2^RNAi^* flies under fed and starved conditions (n = 24). (**F**) Starvation survival of fatbody *dSir2* knockdown (*pSw-S_1_106-gal4>+/+;dSir2^RNAi^ + RU486)* and control (*pSw-S_1_106-gal4>+/+;dSir2^RNAi^ - RU486) flies, fatbody dSir2 knockdown* in *chico heterozygote flies (pSw-S_1_106-gal4>ch*^+/-^; *dSir2^RNAi^ + RU486) and (pSw-S_1_106-gal4>ch*^+/-^; *dSir2^RNAi^- RU486)* (statistical significance indicated in supplementary figure 9) (n = 60).200 μM RU486 was used to knockdown *dSir2* expression. Log Rank was used to plot survival curves and Mantel-Cox test was used for statistical analysis. Student's t-test and ANOVA were used to analyze statistical significance of the data (*, p < 0.05; **, p < 0.01; ***, p < 0.001 or mentioned otherwise).

Insulin-IGF signaling (IIS) is another important nutrient sensing pathway that has been shown to mediate metabolic homeostasis and also affect organismal survival [59, 60]. Therefore, we wanted to check if *dSir^2RNAi^* (+ RU486) flies displayed abrogated IIS. Expression of *dilp* (insulin-like-peptide) genes has been documented to reflect changes in IIS under fed and starved conditions [61]. Using RNA isolated from fly heads, RT-qPCR analyses were performed to quantify the expression dilp-2, -3 and -5 from pSw-tub-gal4> *dSir^2RNAi^* (+ RU486) flies. We found that only *dilp-2* and -5 levels were substantially altered in these flies (Figure 5A and Supplementary Figures 7A and 7B). However, it should be noted that the decrease in *dilp5* expression that was observed during starvation in control flies [61] was absent in *dSir^2RNAi^* (+ RU486) flies (Figure 5A). We also did not see any changes in the expression of *dilp6* levels in the fatbody of control and *dSir^2RNAi^* (+ RU486) flies (Supplementary figure 7C).

Next, we wanted to investigate the contributions of dSir2 activity in the fatbody and muscles in regulating IIS. Interestingly, we found that while knocking down *dSir2* in the fatbody led to an increase in *dilp5* expression, *dSir2* knock down in muscles did not have any effect (Figures 5B and 5C). Further, we observed that dilp5 expression was reduced during starvation in muscle specific*dSir^2RNAi^* (+ RU486) flies similar to controls (Figure 5C), as expected. In contrast, the transcript levels of dilp5 remained high in fatbody specific *dSir^2RNAi^* (+ RU486) flies after 48-hour starvation (Figure 5B).

Since increased *dilp5* expression has been associated with an increase in IIS, we assayed for the expression of insulin receptor and the downstream target gene *d4eBP* in control and fatbody *dSir^2RNAi^* (+ RU486) flies. It is important to note that decreased expression of *d4eBP* is well characterized as a marker of signaling through IIS. When expression of these genes was assayed in fatbody specific *dSir^2RNAi^* (+ RU486) flies, we found that their levels were reminiscent of increased insulin signaling (Figures 5D and 5E).

In order to confirm the role of dSir2 in affecting systemic insulin signaling, we assayed for the expression of *dilp5*, *dInR* and *d4eBP* in *pSw-tub-gal4>Sir2^EP2300^* flies. As shown in Supplementary Figure 8, overexpression of *dSir2* resulted in decreased *dilp5* mediated insulin signaling. Further, these results made us wonder if starvation sensitivity of fatbody *dSir^2RNAi^* (+ RU486) flies was due to increased insulin signaling. To specifically analyze this, we employed *chico* heterozygote flies which exhibit reduced insulin signaling. Importantly, these flies have been shown to outlive control flies when subjected to starvation [62, 63]. To address the effect of fatbody *dSir2* knockdown in the background of *chico* (*ch*) mutation, we generated *pSw-S1106-gal4>*ch^+/-^; *dSir^2RNAi^* flies and administered them with RU486. As reported earlier, chico heterozygotes (*pSw-S1106-gal4>*ch^+/-^; *dSir^2RNAi^* without RU486) [62] displayed starvation resistance when compared to controls. Comparing median and maximal survival showed that flies *pSw-S1106-gal4>*ch^+/-^; *dSir^2RNAi^* with RU486 (*chico* heterozygotes + *dSir2*knockdown) were similar to *pSw-S1106-gal4>*ch^+/-^; *dSir^2RNAi^* with RU486 flies (only *dSir2* knockdown) (Figure 5F and Supplementary Figure 9). Interestingly, we did not observe any significant effect of insulin signaling on starvation survival when *dSir2* was knocked down in the fatbody. These results suggest that reduced starvation resistance in the absence of dSir2 expression is caused by an inability to mobilize energy reserves.

## DISCUSSION

**Figure 6:**
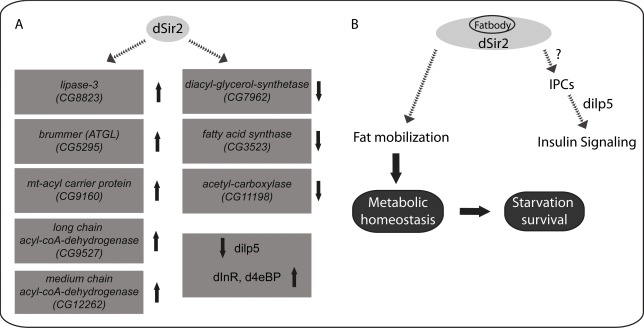
dSir2 regulates metabolic and energy homeostasis and its functions in the fatbody affect systemic insulin signaling

Here, we report that *dSir2* is a critical factor that regulates metabolic homeostasis and mediates organismal physiology (Figure 6). Using genetic tools (inducible RNAi) that negate background effects, we provide concrete results that highlight the importance of endogenous *dSir2* in the whole body, and in metabolically relevant tissues, such as fatbody and muscle. Our findings point out the importance of nutrient signaling in eliciting *dSir2*-dependent molecular changes, which play an important role in tissue specific metabolic functions that affect systemic outputs in flies (Figure 6). By describing a metabolic phenotype in flies that lack *dSir2*, we not only reiterate that Drosophila can be used to study sirtuin biology, but also highlight the evolutionary conservation of dSir2/SIRT1 functions in regulating organismal physiology.

Until now, the conservation of molecular mechanisms underlying *Sir2* biology was poorly addressed in invertebrates. It is only in mammals that a functional interplay between metabolic flux, SIRT1 and its downstream molecular factors has been addressed, thus far [17, 25, 26]. Results from backcrossed *dSir2* mutant and whole body *dSir2* knockdown flies indicated that absence or down-regulation of *dSir2* expression results in gross metabolic defects (Figure 2). Interestingly, we observed that the effects on glucose levels were different in these two cases (Supplementary Figure 3A). The differences in glucose levels might reflect the systemic alterations in response to a complete absence of the protein in the case of mutants and down-regulation of expression in the case of knockdowns. It is interesting to note that studies in *Sirt1^+/-^*, liver specific *Sirt1* knockout [36] and knockdown mice [27, 28] have also yielded seemingly conflicting. Specifically, with respect to glucose metabolism, these differences indicate that the manifestation of functions of Sir2/SIRT1 might be dependent upon the extent to which its expression is altered. Importantly, this underpins the need to further investigate the molecular interactions that bring about such varied phenotypes, in both mammals and flies.

It is important to note that we obtained consistent phenotypic, metabolic and molecular readouts with respect to fat metabolism in *dSir2*-mutant and -RNAi flies. We have found that a decrease (or absence) of *dSir2* expression results in increased fat storage in the fatbodies, as determined by oil red staining and biochemical analyses (Figures 2 and 3). This fat accumulation is due to altered expression of genes involved in fat metabolism (Figures 2 and 3). Importantly, we show that genes involved in fat breakdown are downregulated in the *dSir2* knockdown flies, in addition to an upregulation of genes involved in fat synthesis (Figures 2 and 3). These findings are not only in accordance with the results obtained from *dSir2* mutant larvae [54] but also implicate *dSir2* as a key player in fat metabolism in adult flies.

We have also uncovered a role for *dSir2* in regulating systemic insulin signaling in flies. To investigate if the ability of *dSir2* to mediate insulin signaling emanated from a specific tissue, we assayed for *dilp5* expression in fatbody and muscle specific *dSir2^RNAi^* flies. Interestingly, we have found that knocking down *dSir2* only in the fatbody, but not muscles led to increased dilp5 expression, and mimicked dSir2 mutants and whole body *dSir2^RNAi^* flies (Figure 5 and data not shown). Specifically, we have addressed the role of *dSir2* in the fatbody to mediate systemic effects on insulin signaling (Figure 5 and Supplementary Figure 9). Further investigations should help us understand the dSir2-dependent molecular and physiological links between the fatbody and medial secretory neurons (MSNs). Very recently, hepatic SIRT1 was shown to mediate peripheral insulin signaling in mice [36]. Importantly, our findings underpin the importance of dSir2/SIRT1 in the homologous metabolic tissues, fatbody and liver, on systemic insulin signaling.

Our efforts to link the molecular functions of *dSir2* and organismal physiology led us to implicate *dSir2* in starvation survival. *dSir2* mutants and whole body *dSir2^RNAi^* flies succumb to starvation earlier than the controls and interestingly, are phenocopied by fatbody *dSir2^RNAi^* flies (Figures 1A, 1B and 4A). Moreover we have shown that this is due to an inability to mobilize fat reserves from the fatbody, and a resultant of decreased expression of lipid breakdown genes, both under fed and starved conditions (Figures 2 to 4). The importance of *dSir2* in the fatbody and fat mobilization is corroborated by an absence of deregulated fat metabolism in muscle specific *dSir2^RNAi^* flies (Figure 3). Further, a lack of starvation phenotype when *dSir2* is knocked down from the muscles highlights the physiological relevance of fatbody (Figure 4B).

In summary, we have elucidated the significance of the functions of *dSir2* in the fatbody in mediating central and peripheral effects on metabolic homeostasis and insulin signaling (Figure 6). Therefore, we conclude that *dSir2* is a key component that links dietary inputs with organismal physiology and survival (Figure 6). Most importantly, our study highlights the functions of *dSir2* in the fatbody as a deterministic factor in governing fly physiology. This study delineates the functions of *dSir2* in two metabolic tissues in affecting organismal survival. Metabolic homeostasis and the ability to utilize stored energy reserves are also crucial for mediating the effects of calorie restriction. We believe that our results, which emphasize the importance of dSir2 in maintaining homeostasis reiterates its role in calorie restriction. Finally, our report highlights the need to further investigate the functions of*dSir2*, and should motivate future studies to understanding Sir2's interactions with other pathways and importance during aging.

## METHODS

### Fly Strains

*w^1118^*, *Sir^22A.7.11^*,*Sir2^EP2300^*, cn[1] P{ry[+t7.2]=ry11}chico[1]/CyO;ry[506], and P{Switch1}106-gal4 were obtained from Bloomington Stock Center (Indiana University, USA). P{Switch-tubulin}-gal4 was a kind gift from Dr. Stephen Helfand's Lab. The rp298; P{Switch-MHC}-gal4 was obtained from NCBS, Bangalore, India. The *Sir^22A.7.11^*stock was backcrossed with *w^1118^* for 10 generations before they were used for the assays.dSir2^RNAi^ (CG5216:23201/GD) was obtained from the Vienna Drosophila RNAi Center (VDRC). Flies were grown on normal food under non-crowding conditions at 25°C with 12/12h light/dark cycle. Age-matched virgin female flies were used for this study.

### Fly Diets

Following different dietary regimes were used for metabolic alterations [44]. Normal Diet (ND) contained yeast extract (2.5%), sucrose (5%) and cornmeal (8.6%). For low yeast medium, yeast extract was reduced to 0.25%, and for high yeast medium yeast extract was increased to 5%, keeping all the other components the same.

### Starvation Assay

Three-day-old flies were kept in vials (10 per vial) containing 2% agar and transferred into fresh vials every 8 hours. To determine starvation survival response, dead flies were scored after every transfer. To characterize metabolic and molecular effects of starvation flies were starved (as mentioned earlier) for 48 hours and snap frozen in liquid nitrogen.

### Activation of inducible gal4

The inducible gal4 (PSwitch lines) was activated by rearing flies on medium containing 200 μM RU486 (Mifepristone; Sigma Cat No.M8046) (in ethanol). Flies reared on a diet containing only ethanol were used as controls.

### RNA isolation and Reverse Transcription

Total RNA was isolated from 8 flies (pooled together) using Trizol (Invitrogen, Cat. no. 15596-026) according to the manufacturer's instructions. 1μg of RNA was used for cDNA synthesis using SSIII reverse transcriptase kit (Invitrogen, Cat. no. 18080-044) as per the manufacturer's instructions.

### Real time PCR analyses

qPCR was performed using Quantifast SYBR green (Qiagen, Cat. no. 204054) and Eppendorf Realplex instrument. The cycling conditions were as prescribed by the manufacturer. The primer pairs used for the assay are tabulated in Supplementary Table 1. Levels of Actin (actin5c) were used for normalization.

### Lipid and Glucose Measurement

Six (3-5 day old) flies reared on normal diet were snap frozen in liquid nitrogen and homogenized in 600 μL PBST (PBS with 0.05% Tween-20). The homogenate was heated to 70°C for 10 minutes. Following centrifugation at 12000 rpm for 15 minutes, the supernatant was transferred to fresh tubes and were assayed at a pathological laboratory (Shahbazker's Lab, Colaba Mumbai-400005).

### Oil-Red staining of fat bodies

Fat bodies from adult flies were dissected out and fixed in 4% formaldehyde. Followed by washing with running tap water for 10 minutes, the fixed samples were stained with freshly prepared 60% Oil Red O (Sigma Aldrich Cat. # 0625-100G) solution for 20 minutes and then rinsed with 60% isopropanol and PBS. The samples were mounted onto a slide and imaged using LSM-510 Carl Zeiss microscope.

### Western Blot

Lysates were prepared, by incubating on ice for 15-20 minutes, in a buffer containing 50mM Tris-HCl pH7.5, 150mM NaCl, 1mM EDTA, 6mM EGTA, 20mM NaF, 1% Triton X-100, and protease inhibitors (Roche, Cat no. 05-056-489001). The lysates were then centrifuged at 12,000 rpm for 15 minutes at 4C to pellet the debris. Supernatants were used for protein estimations using BCA kit (Sigma, Cat no. BCA9643) and resolved on a 10% SDS-PAGE. Following electrotransfer onto PVDF membranes they were probed with appropriate antibodies according to standard procedures. Anti-Sir2 (sc-300) and anti-Actin antibodies were purchased from Santa Cruz Biotechnology (Cat. no. Sc-98262) and Sigma Aldrich (Cat. no. A1978), respectively. Chemiluminescence detection (Roche Cat. no. 12-015-196001) was used to visualize the bands.

### NAD Assay

NAD^+^ was extracted as explained by McElfresh and McDonald [45]. Briefly, 8 flies were homogenized in 1mL of 0.5M Perchloric Acid (PCA) and centrifuged at 10, 000g for 3 minutes. The supernatant was treated with equal volumes of 1M potassium phosphate and neutralized with 3N KOH followed by 2 hours incubation at 4°C. To 350 μl of neutralized extracts, 310 μl of reaction buffer (0.232M Bicine, 2mg/ml BSA, 1.16M ethanol, 9.67mM EDTA, 3.87mM phenazineethosulphate, 1mM MTT) was added and incubated at 37°C for 5 minutes. Then cycling assay was initiated by the addition of 60μl of 0.6mg/ml alcohol dehydrogenase. After 30 minutes adding 300μl of 12mM iodoacetate terminated the reaction and the absorbance was read at 570nm. Total NAD^+^ was normalized to protein content.

### Statistical analyses

Graphpad Instat3 and SigmaPlot were used for all statistical analyses. Log Rank was used to plot survival curves and Mantel-Cox test was used for statistical analysis. Student's t-test and ANOVA were used to analyze statistical significance of the data (*, p < 0.05; **, p < 0.01; ***, p < 0.001 or mentioned otherwise).

## SUPPLEMENTARY FIGURES

Supplementary Figure 1

Supplementary Figure 2

Supplementary Figure 3

Supplementary Figure 4

Supplementary Figure 5

Supplementary Figure 6

Supplementary Figure 7

Supplementary Figure 8

Supplementary Figure 9

Supplementary Table 1

## Abbreviations

ST: starvation
ND: normal diet
