# Supplementary figures and images for: dSir2 deficiency in the fatbody, but not muscles, affects systemic insulin signaling, fat mobilization and starvation survival in flies
Source: Aging (Albany NY). 2012 Mar 10;4(3):206–23. doi: 10.18632/aging.100435 (PMC3348481; doi:10.18632/aging.100435)

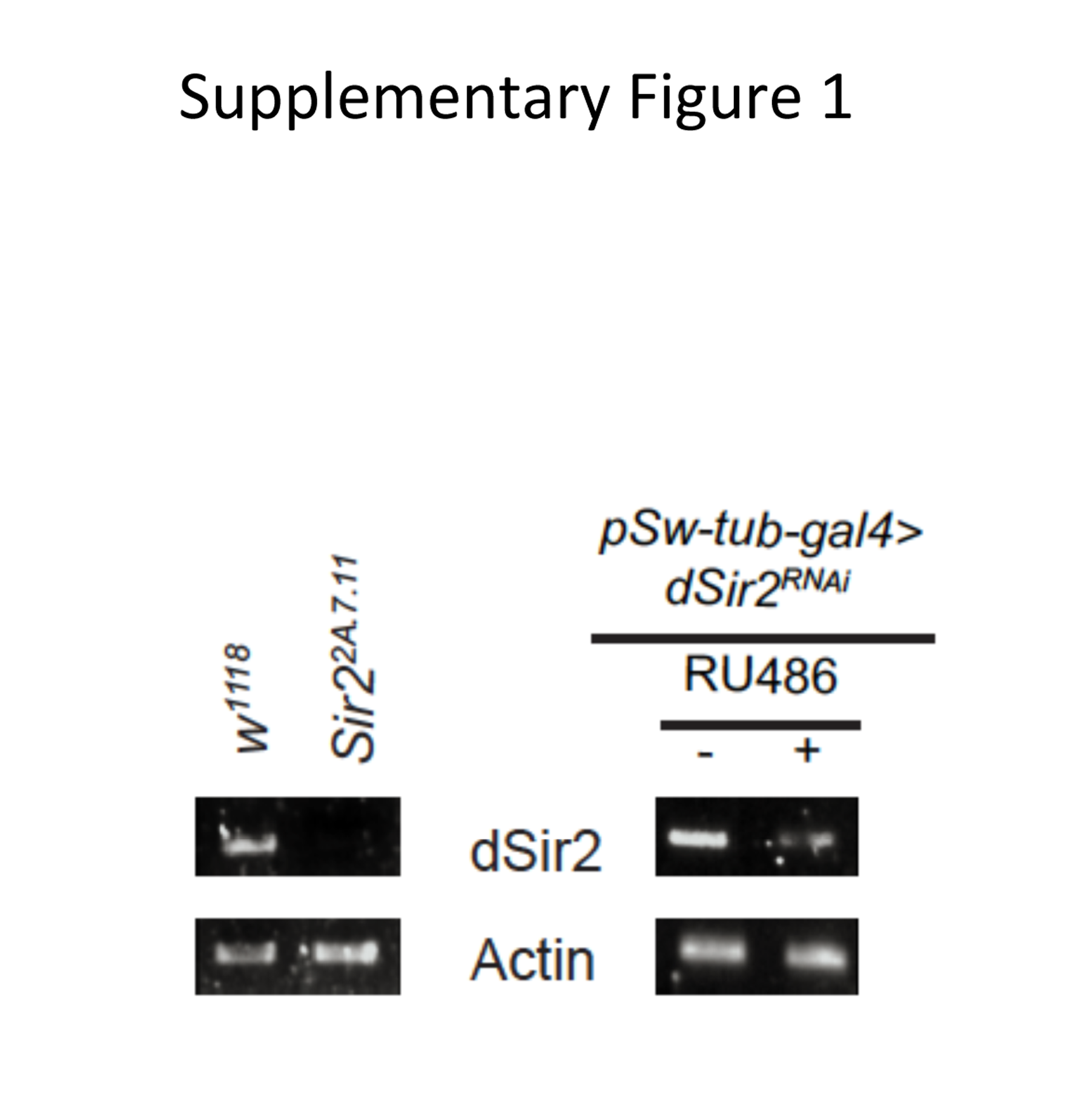

Supplement: Supplementary Figure 1 [file aging-04-206-s001.tif]

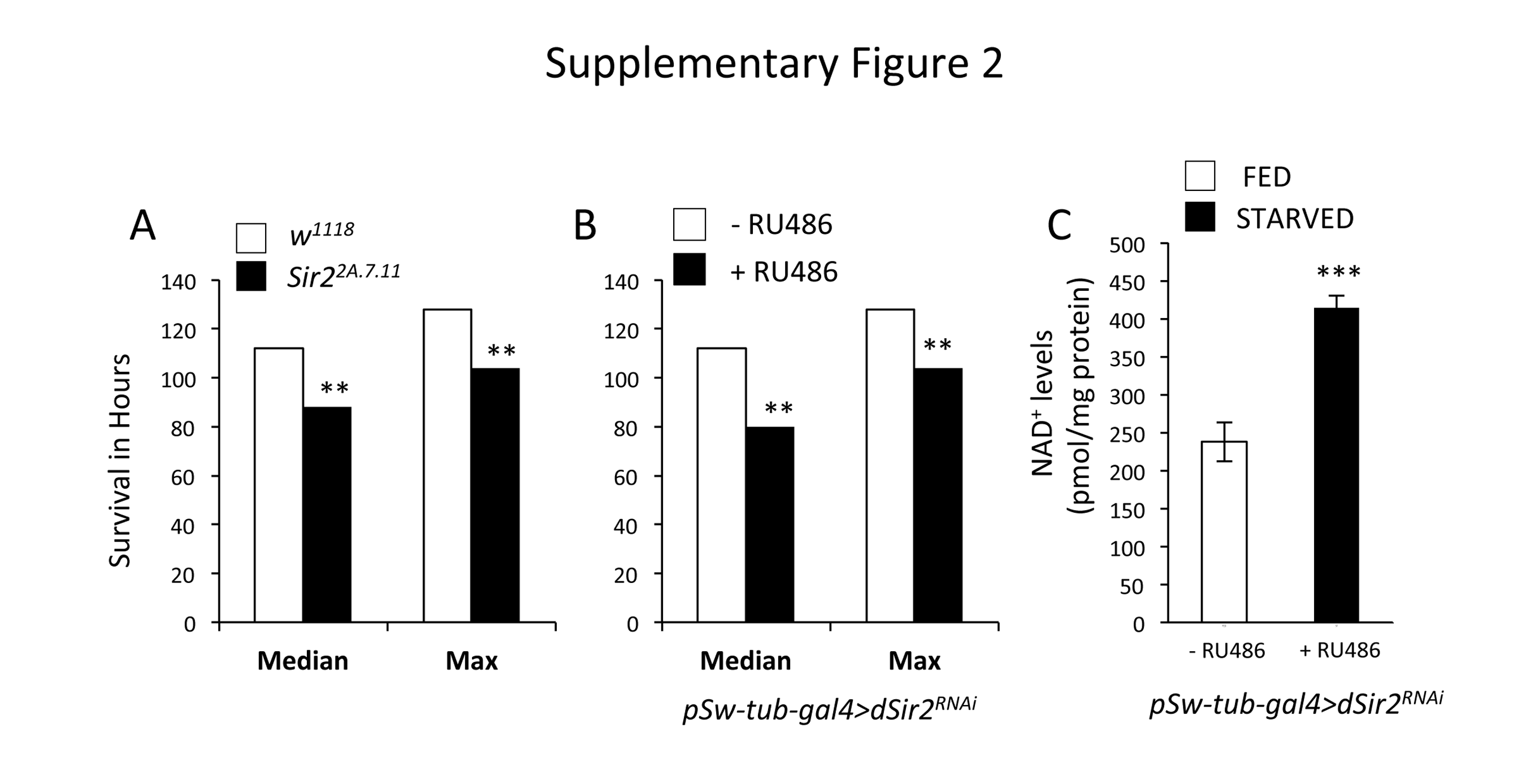

Supplement: Supplementary Figure 2 [file aging-04-206-s002.tif]

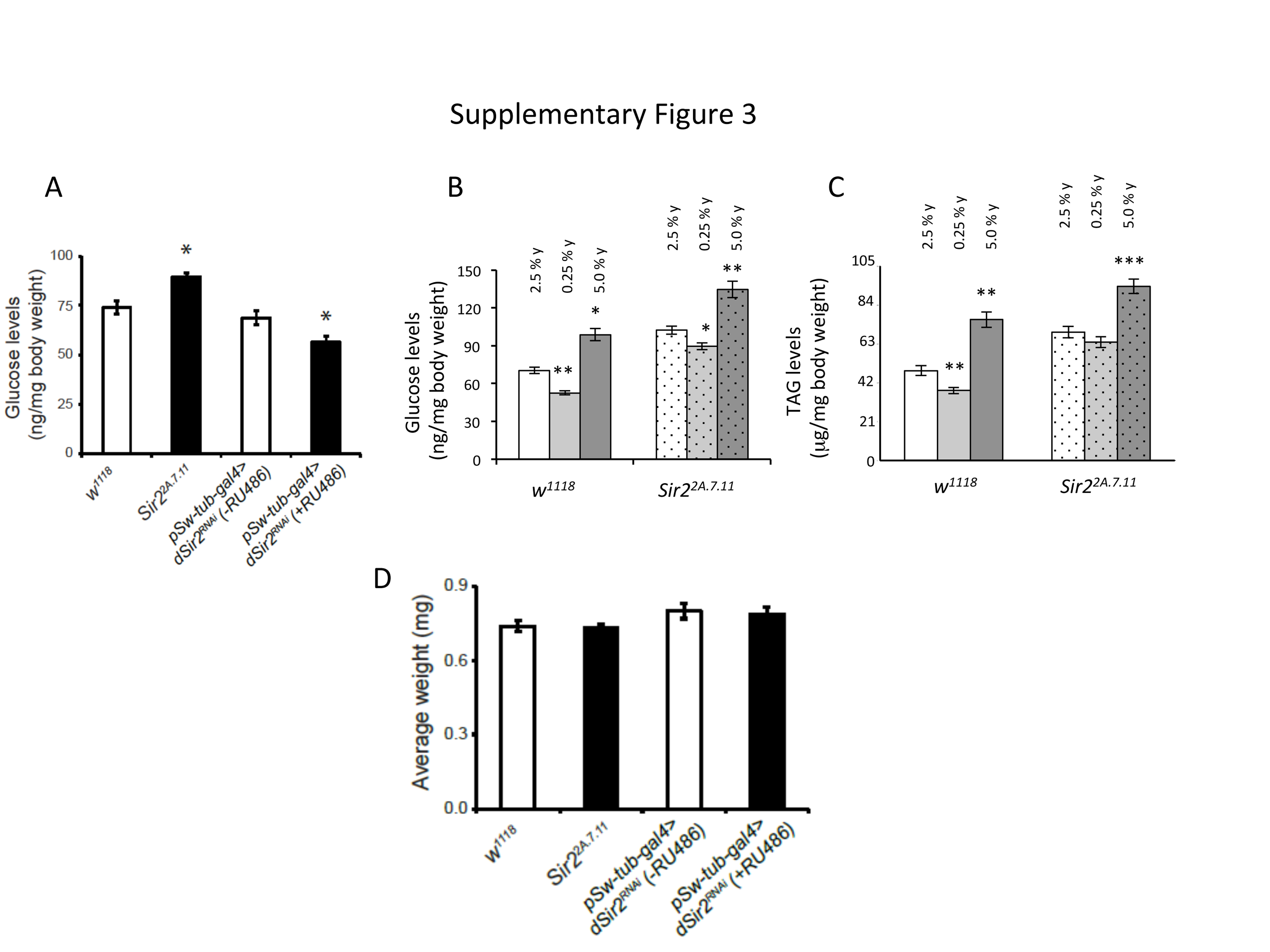

Supplement: Supplementary Figure 3 [file aging-04-206-s003.tif]

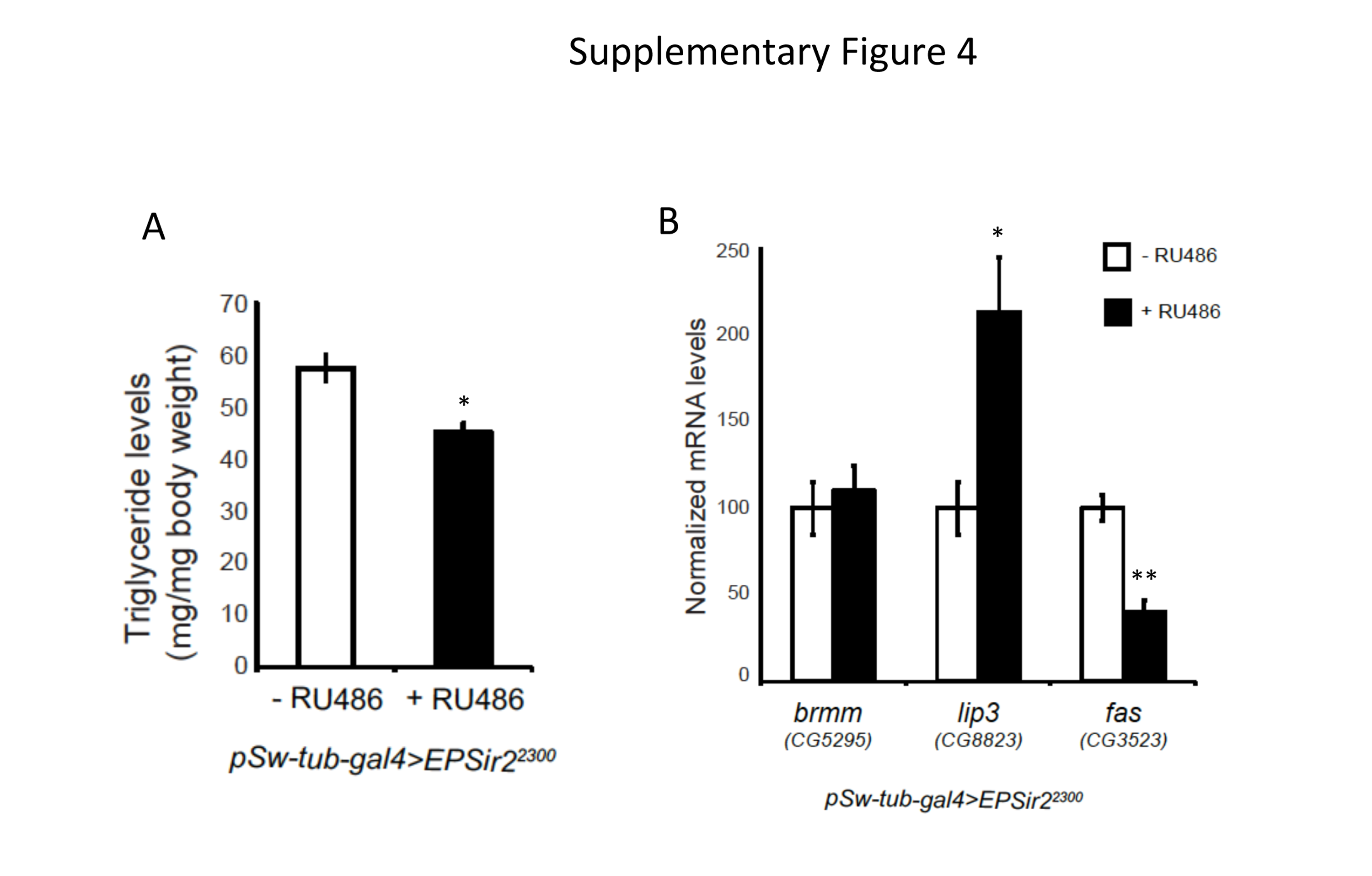

Supplement: Supplementary Figure 4 [file aging-04-206-s004.tif]

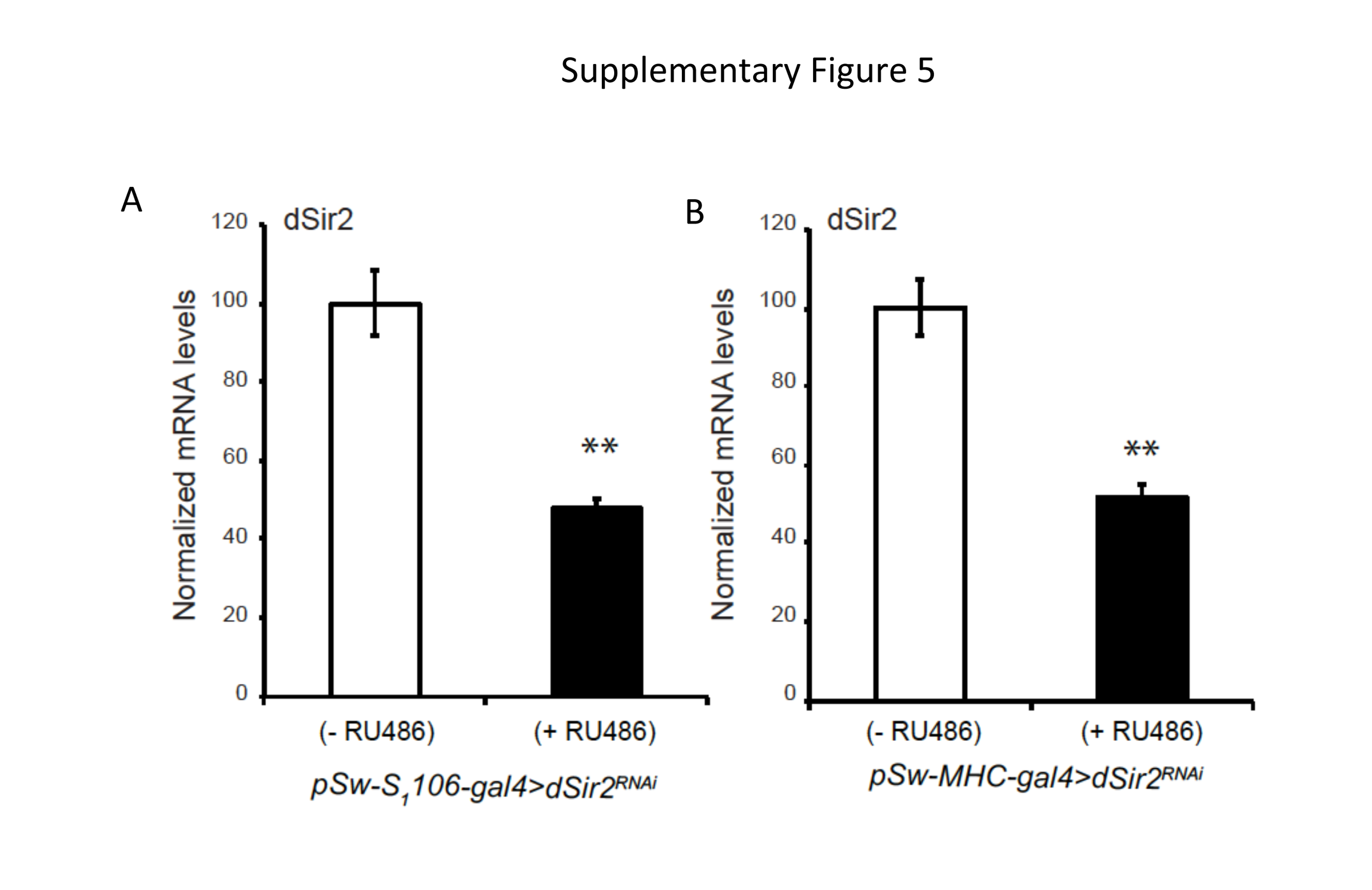

Supplement: Supplementary Figure 5 [file aging-04-206-s005.tif]

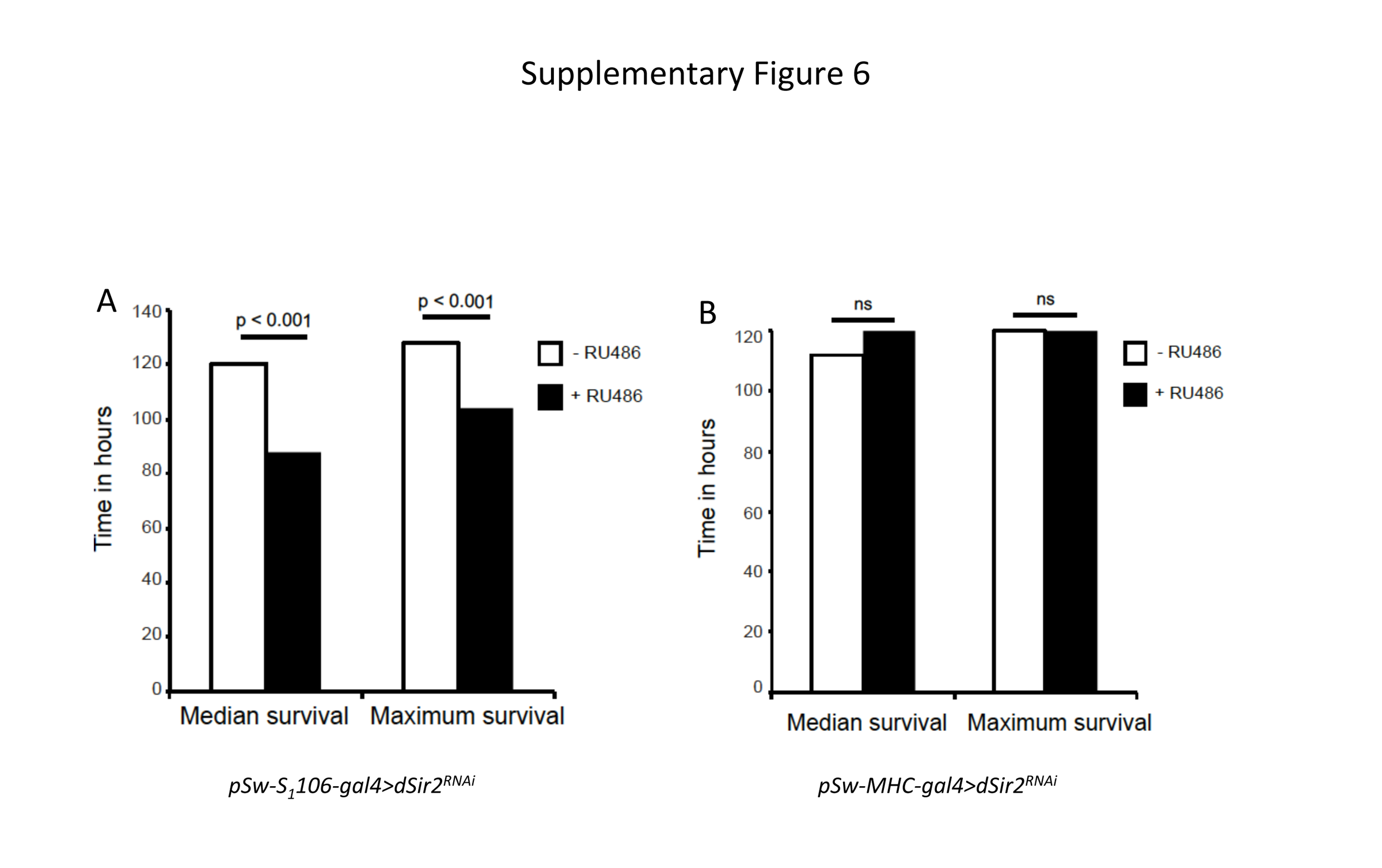

Supplement: Supplementary Figure 6 [file aging-04-206-s006.tif]

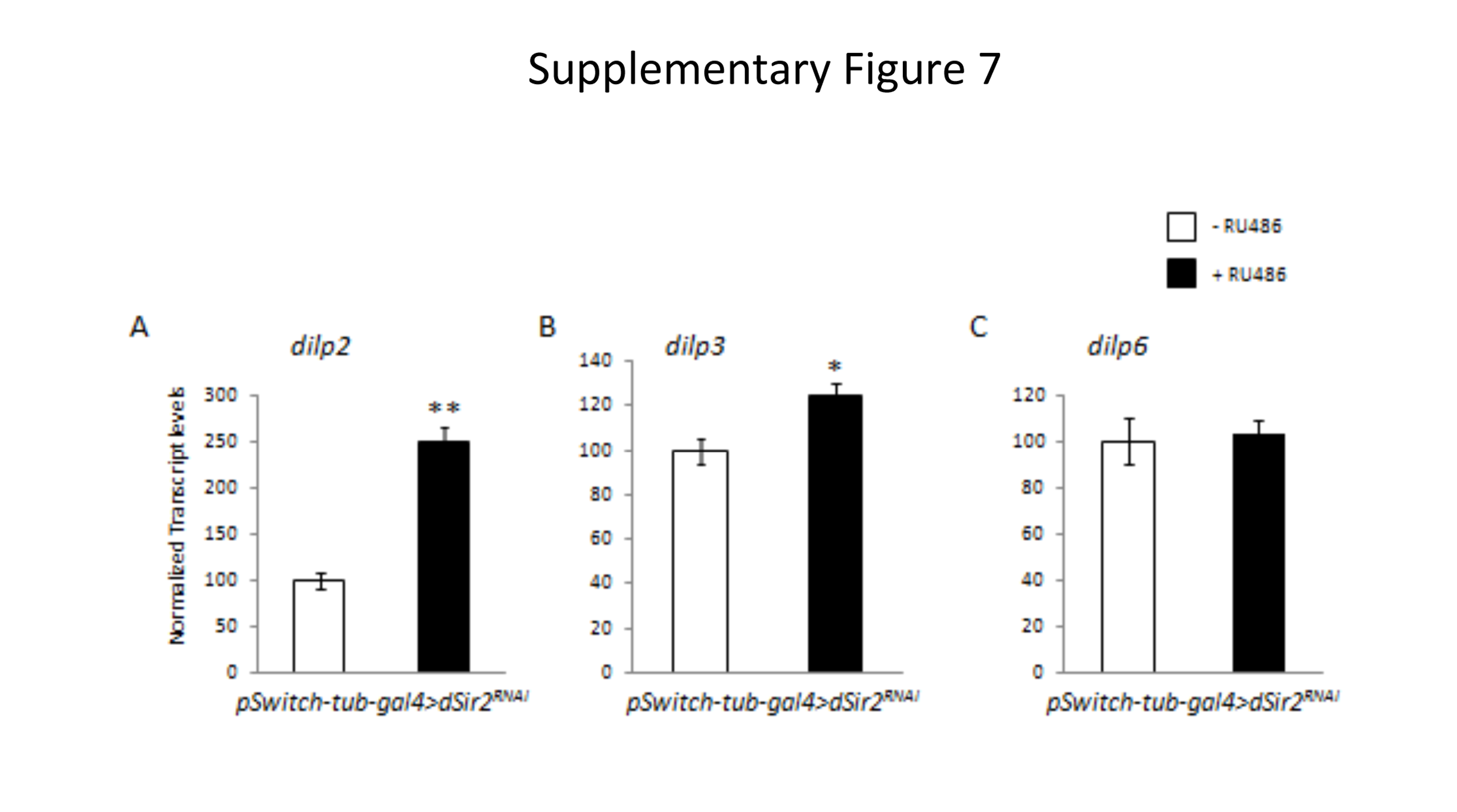

Supplement: Supplementary Figure 7 [file aging-04-206-s007.tif]

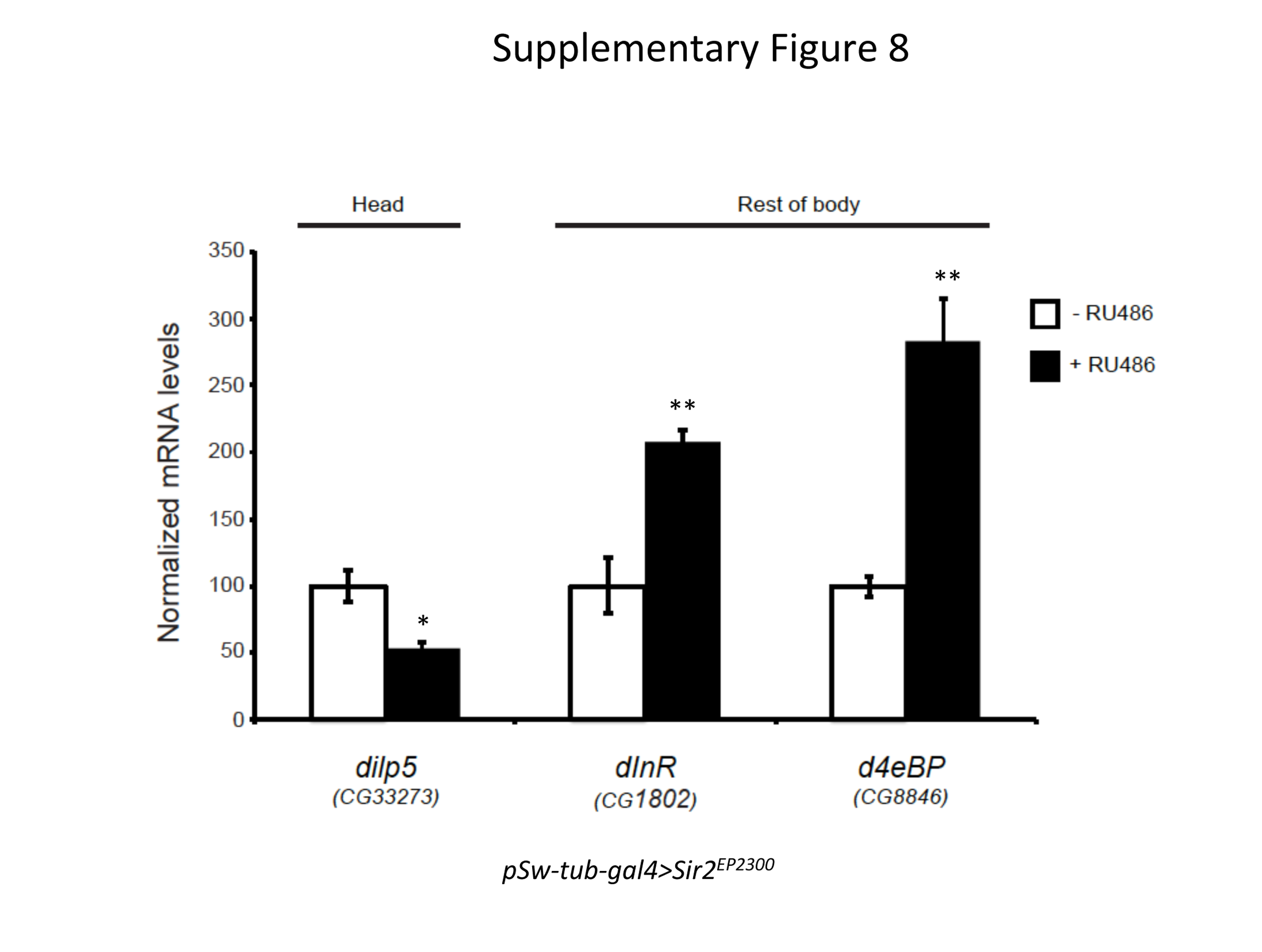

Supplement: Supplementary Figure 8 [file aging-04-206-s008.tif]

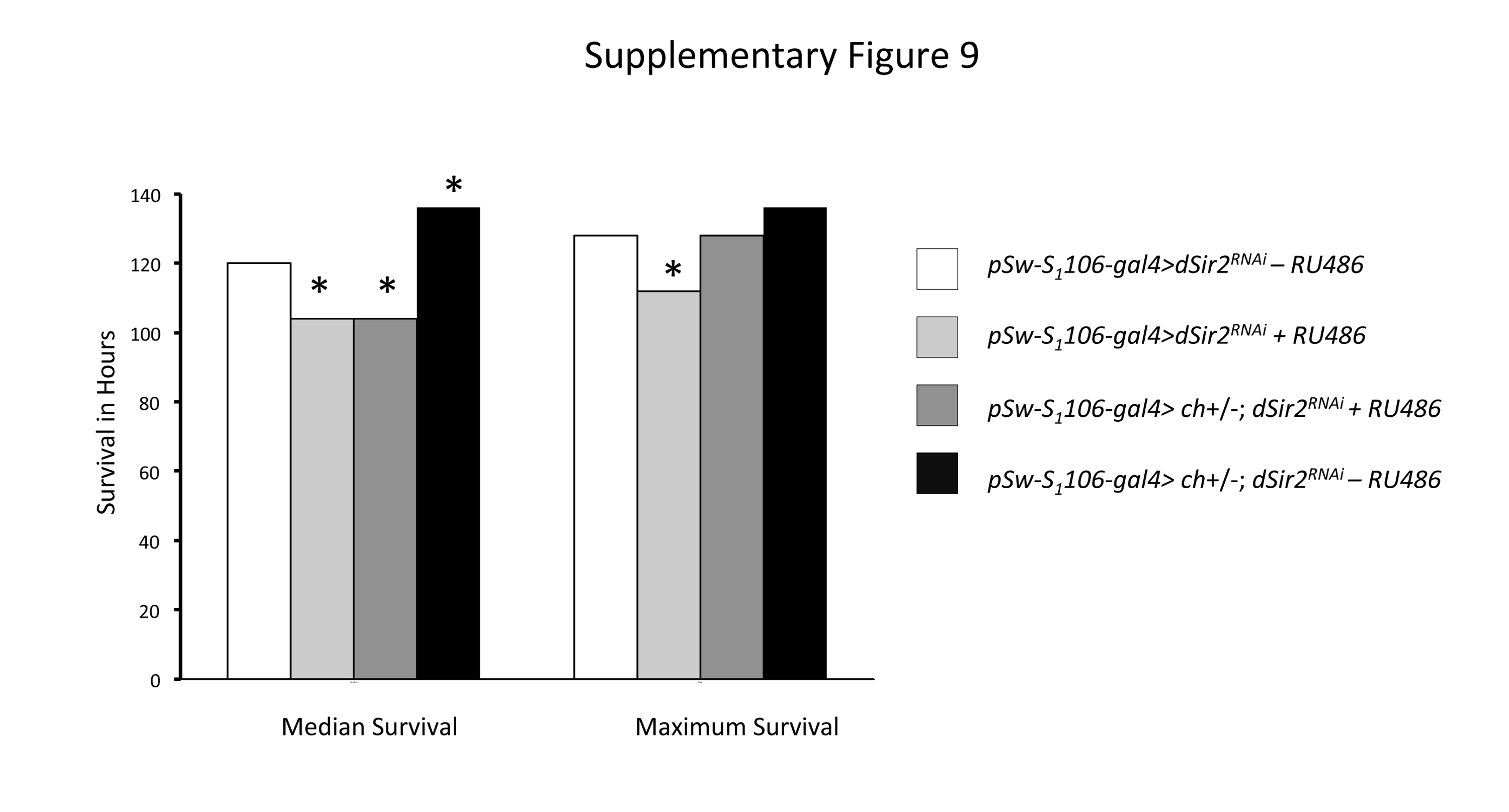

Supplement: Supplementary Figure 9 [file aging-04-206-s009.tif]

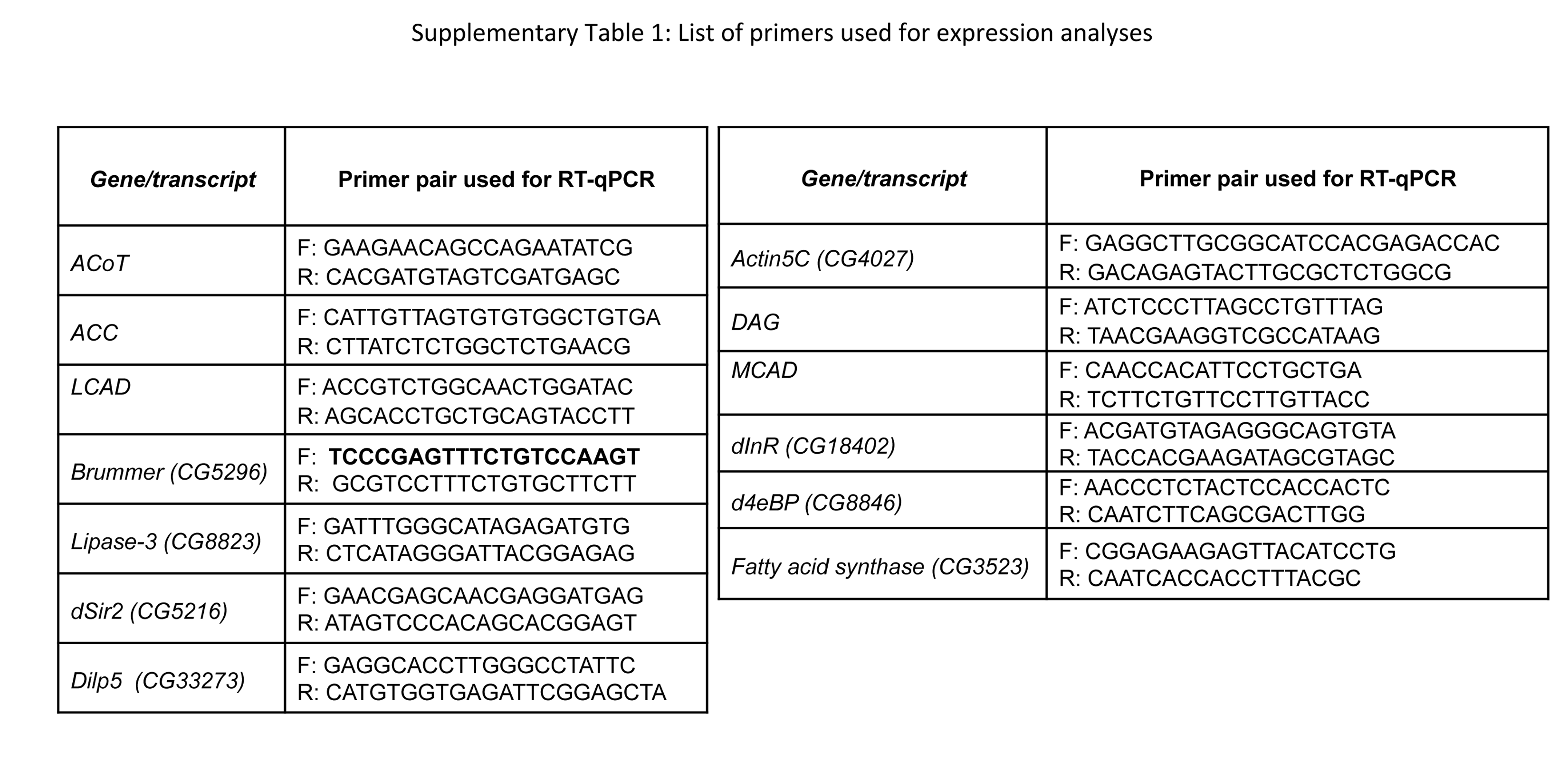

Supplement: Supplementary Table 1 [file aging-04-206-s010.tif]
